# The face inversion effect in opponent-stimulus rivalry

**DOI:** 10.3389/fnhum.2014.00295

**Published:** 2014-05-15

**Authors:** Malte Persike, Bozana Meinhardt-Injac, Günter Meinhardt

**Affiliations:** Research Methods and Statistics, Department of Psychology, Institute of Psychology, Johannes Gutenberg University MainzMainz, Germany

**Keywords:** binocular rivalry, inversion effect, visual awareness, predominance ratio, face specificity

## Abstract

The face inversion effect is regarded as a hallmark of face-specific processing, and can be observed in a large variety of visual tasks. Face inversion effects are also reported in binocular rivalry. However, it is unclear whether these effects are face-specific, and distinct from the general tendency of visual awareness to privilege upright objects. We studied continuous rivalry across more than 600 dominance epochs for each observer, having faces and houses rival against their inverted counterparts, and letting faces rival against houses in both upright and inverted orientation. We found strong inversion effects for faces and houses in both the frequency of dominance epochs and their duration. Inversion effects for faces, however, were substantially larger, reaching a 70:30 distribution of dominance times for upright versus inverted faces, while a 60:40 distribution was obtained for upright versus inverted houses. Inversion effects for faces reached a Cohen's *d* of 0.85, compared to a value of 0.33 for houses. Dominance times for rivalry of faces against houses had a 60:40 distribution in favor of faces, independent of the orientation of the objects. These results confirm the general tendency of visual awareness to prefer upright objects, and demonstrate the outstanding role of faces. Since effect size measures clearly distinguish face stimuli in opponent-stimulus rivalry, the method is highly recommended for testing the effects of face manipulations against non-face reference objects.

## 1. Introduction

When presenting highly dissimilar images to corresponding regions of either eye an observer experiences binocular rivalry—dynamic alternations of two percepts that compete for dominance. Because the physical stimuli are constantly visible to each eye but conscious perception fluctuates, binocular rivalry ranks among the most intriguing paradigms to study properties of visual awareness. While in earlier conceptualizations it was proposed that binocular rivalry reflected competition between monocular neurons within the LGN and the primary visual cortex (Blake, 1989), it has since been established that competitive interactions at multiple neural sites are involved, including lower and eye-specific, and also higher cortical areas which respond to input from both eyes (Blake and Logothetis, 2002; Tong et al., 2006). Although the issue is still subject to ongoing debate, the involvement of higher, object related cortical levels with input from both eyes has contributed to the idea that neural representations of the two stimuli compete for visual awareness, independent of the eye that actually views the stimulus (Leopold and Logothetis, 1996; Logothetis et al., 1996). A striking observation in favor of pattern competition rather than eye competition was that subjects experienced no dominance changes when sudden eye-reversals of stimulus presentations were introduced in flickering displays (Logothetis et al., 1996), suggesting that eye-independent mechanisms stabilize the conscious experience of the dominant stimulus alternative.

Evidence for pattern competition was mostly found with complex object stimuli which particularly stimulate extrastriate, object related brain regions lacking retinotopic organization and responding largely independent of scale or viewpoint. Using dichoptic presentation of face and house stimuli it was found that activation in the face-tuned fusiform face area (FFA; Kanwisher and Yovel, 2006) alternated with activation in the parahippocampal place area (PPA), which preferably responds to houses and places (Tong et al., 1998), in the same way as if the two single eyes were stimulated with faces and houses in physical alternation. Exploring the remainder FFA activity during the epochs where the perception of intact face stimuli was suppressed it was found that this activity was still greater than the activity caused by invisible scrambled faces (Jiang and He, 2006). This suggests that stimulus processing still reaches higher level areas even if conscious perception is suppressed (Tong et al., 2006).

Earlier studies on binocular rivalry reported influence of object-related, configural stimulus properties. Controlling for low level stimulus properties, faces were still found to have stronger dominance phases compared to random dot patterns (Yu and Blake, 1992). The authors moreover found stronger dominance for dot patterns that could be grouped to meaningful structures (“dalmatian dog”) compared to random patterns that lacked this property. Surprisingly, the advantage for the dalmatian dog patterns was found irrespective of whether the subjects had consciously recognized the structure as meaningful, or not. These and related observations support the notion that activity from higher level visual areas rather than adaptation of eye-tuned neurons during their mutual inhibition initiates the perceptual switch among the rivaling percepts.

Yu and Blake (1992) also reported an advantage of upright orientation over inverted presentation for meaningful dot patterns. Such inversion effects in binocular rivalry suggest that familiarity and learning history with common objects influence their time of conscious perception and suppression (Jiang et al., 2007). Inversion effects play a particular role in face perception, since faces are the object category whose correct perceptual assessment depends strongest on the upright orientation (Yin, 1969). Humans are face experts, and can recognize faces correctly even from distorted images, unusual viewpoints, or after significant aging, unless they are turned upside down (Maurer et al., 2002). Even strong distortions, which make a face appear grotesque, remain unnoticed when a face is turned upside-down (“Thatcher illusion”; Thompson, 1980). These observations led to the conclusion that inversion mainly affects processing of the configural properties of faces, while featural properties remain relatively unaffected by inversion (Carey and Diamond, 1977; Murray et al., 2000; Leder et al., 2001). However, there are also claims that the same facial cues are used for upright and inverted faces (Sekuler et al., 2004), and that inversion effects are not different for single features or features in the usual facial configuration (Rakover and Teucher, 1997), leading to a debate whether inversion changes face processing qualitatively (Rossion and Boremanse, 2008) or quantitatively (Riesenhuber et al., 2004; Sekuler et al., 2004; Riesenhuber and Wolff, 2009). However, measures of holistic face perception, such as the part-whole effect (Tanaka and Farah, 1993) and the composite effect (Young et al., 1987), are likewise critically dependent on the upright orientation (Rossion and Boremanse, 2008). Meanwhile, the face inversion effect (FIE) is recognized as one important hallmark of face speciality, and FIE measurement is used whenever the involvement of proprietary face-specific mechanisms is investigated (Maurer et al., 2002).

In binocular rivalry, early evidence for predominance of upright compared to inverted faces was reported by Engel (1959) who asked subjects to give a summary statement about predominance over a fixed epoch of 1 min length. Using a novel variant of binocular rivalry termed continuous flash suppression (CFS; Tsuchiya and Koch, 2005), Jiang and colleagues showed that upright faces break predominance of dynamic noise patterns in the first rival epoch about 400 ms earlier than inverted faces (Jiang et al., 2007). However, no further control objects were used to indicate whether the upright advantage of faces is face-specific. Using the same paradigm and adding house control objects Zhou et al. (2010) replicated the FIE. Upright faces broke the first dominance epoch of noise patterns earlier than inverted faces, while identical durations were obtained for upright versus inverted houses, indicating face specificity of the inversion effect in the CFS paradigm. A recent CFS study with objects from a variety of categories, however, amended this finding (Stein et al., 2012). The authors reported inversion effects for bodies, faces, dogs, and birds, but no or minor ones for lamps and chairs. Using a relative change measure to normalize the effects they documented disproportionately large inversion effects for faces and bodies, indicating that these two object categories are largely separated in terms of the strength of the inversion effect.

The results of Stein and colleagues are promising for using CFS as a paradigm to identify face-specific effects when contrasted with object categories which are analyzed in a part-based fashion, like houses (Yovel and Kanwisher, 2004; Kanwisher and Yovel, 2006) or cars (Cassia et al., 2009). Interestingly, recent reports of face inversion effects all stem from the CFS paradigm (Yang et al., 2007; Stein et al., 2011a,b, 2012). With the traditional opponent-stimulus rivalry paradigm there are currently no data on the inversion effect for faces compared to other objects categories. The current study aims at filling this gap by systematically comparing inversion effects for faces and houses, since houses are preferably chosen as non-face reference objects in neuroimaging studies on face perception. By estimating effect size measures strength and object specificity of inversion effects observed in CFS and opponent-stimulus rivalry can be directly compared. This may offer a offers a basis for deciding which rivalry paradigm is more appropriate for testing a given set of hypotheses.

## 2. Materials and methods

### 2.1. Study outline

The study aimed at measuring the effects of stimulus inversion for face and house stimuli in opponent-stimulus rivalry. In experiment I faces and houses rivaled against their inverted counterparts. In experiment II faces rivaled against houses, both in upright and inverted orientation. Eye-reversal and artificial blink events were included to indicate eye- or pattern dominance (Blake et al., 1980; Logothetis et al., 1996). Experimental sessions were executed on four consecutive days to obtain representative within-subject data allowing to generalize over temporal state variations between days. Each session comprised four experimental runs for each of the four stimulus conditions. Since comparison of dominance and suppression across stimulus categories requires a match in low level stimulus properties (Yu and Blake, 1992) we conformed the stimulus material with respect to their spatial dimensions and RMS contrast (Peli, 1990). The latter was achieved via an image manipulation procedure that produced images with identical gray level histograms (see below). Hence, the stimulus material matched not only in gray-level variance, but also in its first order image statistics. Since the proportion of mixed dominance epochs, where subjects could not decide whether stimulus alternative A or B was dominant, increases with image size (Yu and Blake, 1992) we adjusted image size such that not more than 50% of mixed dominance epochs could be expected, while the images were still sufficiently large to contain the relevant object details. This also provided leeway to obtain effects pertaining to each rival alternative and the mixed percept. Dominance was measured in terms of epoch frequency, duration, and their joint effect. Effect sizes and normalized effect measures were estimated.

### 2.2. Participants

Seventeen German volunteers participated in this study (12 females and 5 males). All were undergraduate students of psychology at the Johannes Gutenberg University Mainz, age span 20–24 years. All participants had normal or corrected to normal vision, using corrective lenses in the latter case. All subjects were naive with respect to the purpose of the experiment. They were given course credit points for participation. The study was conducted in accordance with the Declaration of Helsinki. In detail, subjects participated voluntarily and gave written informed consent to their participation. In addition, participants were informed that they were free to stop the experiment at any time without negative consequences. The data were analyzed anonymously.

### 2.3. Apparatus

The experiment was executed on standard desktop computers with Inquisit 4 runtime units. Subjects viewed dichoptically through a custom built mirror stereoscope from a viewing distance of 60 cm. Responses were given via external Cedrus RB-830 response pads with internal high-precision timers for accurate response time measurements. Patterns were displayed on NEC MultiSync E222W TFT displays at 1650 × 1050 pixel resolution and a refresh rate of 60 Hz. No gamma correction was used. The room was darkened so that the ambient illumination approximately matched the illumination on the screen.

### 2.4. Stimuli

**Figure 1:**
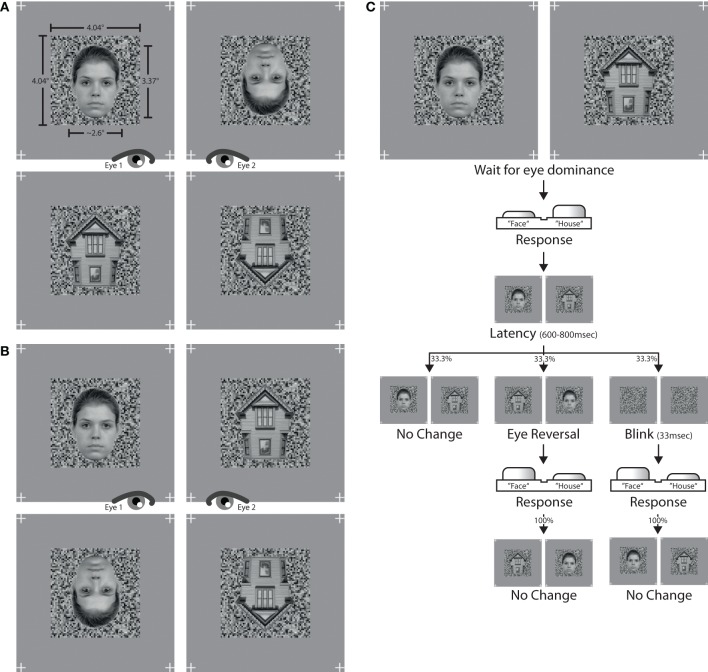
**Stimuli and trial**. The left panel shows the stimulus combinations used in **(A)** experiment I and **(B)** experiment II. The assignment of stimulus to eye altered over the course of an experiment. The right panel **(C)** depicts the trial sequence used in both experiments.

Photographs of faces and houses were selected as stimulus patterns. Face images were selected from the Radboud Faces Database (Langner et al., 2012), house images were sampled from internet sources. Faces were frontal views of eight caucasian models with neutral facial expression. House photographs were eight straight shots depicting the gable end of the structure (see Figure 1). Picture backgrounds were removed in Adobe Photoshop. The images were converted to grayscale and downsampled to a picture height of 125 pixels, or 3.37° of visual angle. The widths of both faces and houses spanned from 90 to 110 pixels, or 2.42° to 2.96°, depending on the specific aspect proportions of a given image. To achieve maximal congruency in pixel overlap between two dichoptically presented images, pairs of face and house images with similar shape and geometry were assembled. Only these matching pairs of faces and houses were set against each other in the experiment. Images were flipped over the horizontal axis to create inverted versions.

Luminance histograms of all images were equalized with Matlab procedures developed in-house. First, the average histogram of pixel intensity values was computed across all images. An adaptive quantile transformation then conformed the pixel intensities of each image to the average histogram, yielding images with identical luminance histograms. The mean luminance of each image was 0.518 in a normalized [0,1] range, or 93.2 cd/m^2^ on screen. Maximum screen luminance was 187.7 cd/m^2^ and minimum screen luminance was 3.7 cd/m^2^. RMS contrast (Peli, 1990) of all images was 0.176 in normalized units. Images were finally superimposed onto a background noise pattern with a size of 150 × 150 pixels, or 4.04° × 4.04°, and a grain resolution of three pixels. The luminance distribution of the noise pattern was sampled from the previously computed average luminance histogram in order to keep the luminance distribution of the whole stimulus unchanged. The background pattern was identical for both eyes and only changed between experimental conditions. This was done to help observers maintain eye vergence on the whole stimulus during foreground changes. In addition, four location markers were placed right outside the corners of the background pattern at positions identical to each eye. The whole stimulus arrangement was displayed on a gray screen canvas with a luminance of 93.2 cd/m^2^, thereby matching the mean luminance of each stimulus. See Figure 1 for stimulus examples from experiment I (Figure 1A) and experiment II (Figure 1B).

### 2.5. Procedure

Prior to each experimental session, participants completed an extensive calibration procedure to adjust the stereoscope to their ocular anatomy and vergence disposition. In addition, a standard blink test was performed to determine the dominant eye. Fifteen of the seventeen participants were right-dominant.

The main blocks of both experiments comprised two stimulus conditions, constructed from different pairings of stimuli. Experiment 1 contained pairings of (a) upright faces with inverted faces, and (b) upright houses with inverted houses. Experiment 2 paired (a) upright faces with upright houses, and (b) inverted faces with inverted houses. Figure 1 provides stimulus examples for all stimulus conditions from both experiments. Since each experimental condition presented different stimulus types, the assignment of response button to stimulus category needed to be learned before entering the main experimental block. The learning task consisted of 64 trials, 32 trials for each of the two stimulus categories which were to be juxtaposed in the main experiment. A learning trial was the binocular display of one stimulus, viewed through the stereoscope. Participants had to press the response button corresponding to the stimulus category on screen. Participants were allowed to proceed to the main experiment only if they reached a proportion correct rate of at least 0.96, i.e., no more than 2 errors in 64 learning trials.

A main experimental block started with the dichoptic display of one stimulus pair (Figure 1C). Subjects indicated via a button press which of the two stimuli was perceived as unambiguously dominant at any given moment. When none of the two stimuli was dominant, thus resulting in a fused percept containing parts of both stimuli, both response buttons were to be released. A button press was followed by a latency period of 600–800 ms allowing for the dominance percept to consolidate. If the button was released while still within latency, no experimental manipulation commenced. If, however, the button press was retained until after the latency period, one of three experimental manipulations took effect with equal likelihood. First, the stimulus presentation could remain unaltered by keeping the same stimulus arrangement on screen as before the button press (the “no-change,” or “normal” condition). Second, the stimulus presentation could be reversed between eyes, so that each eye would afterwards be presented with that stimulus which the other eye had viewed before (the “eye reversal” condition). Third, both stimuli could disappear for two frames (33 ms) leaving only the underlying background mask visible, and then reappear in the same stimulus arrangement as before (the “blink” condition). When either an eye reversal or a blink had occurred, the next three button presses never triggered a latency phase but had the respective epoch always be of the no-change variant without any stimulus change. This was done in order to avoid rapid cascades of eye reversals or blinks on consecutive button presses. The procedure further ascertained that about ½ of all epochs were no-change epochs, ¼ eye reversal epochs and ¼ blink epochs.

The four opponent-stimulus rivalry conditions were blocked and administered during one single session. Participants were asked to take brief pauses between experimental blocks. Each participant attended four sessions for the respective experiment over the course of four consecutive days. A session comprised 64 epochs in each learning task and 240 epochs in each experimental block, 180 of which were no-change trials, 30 eye reversal trials, and 30 blink trials. A session took observers between 40 and 60 min. Participants were free to stop the experiment at any given time via an exit button if they felt the task became uncomfortable.

### 2.6. Dependent measures and outlier clearing

The length of dominance epochs was recorded for each stimulus category in both possible pairings (see previous section). A dominance epoch was defined as the time duration for which participants had one of the response buttons depressed. Moreover, the duration of ambiguous epochs was recorded, where participants reported an unclear percept containing parts from both presented stimuli. Note that pairwise stimulus rivalry, as employed here, may yield different dominance durations for the same stimulus category, depending on which other stimulus it is paired with. Hence, each of the four stimulus conditions produces two sets of dominance durations. For example, dominance durations for the “upright face” category can either stem from its paring with inverted faces or upright houses.

For each subject the data from all four sessions per stimulus condition were merged into one data set. Since response time measurements are susceptible to lapses in attention and erroneously prolonged button presses, dominance durations were cleared for outliers by calculating the mean (*M*) and standard deviation (*SD*) for each set of dominance epochs and clipping all dominance durations beyond *M* + 2.5*SD*. For no participant, more than 1.94% of the recorded dominance epochs were excluded. The raw data of all subjects, including the positions of the outlier criteria on the time continuum, are supplied in the electronic supplement of this article.

### 2.7. Data analysis

The frequency of dominance epochs and their duration were analyzed with repeated measurement ANOVA. Separate analyses were carried out for each experiment and each dependent variable. The data of experiment I were analyzed for effects of percept (upright or inverted), object type (face or house) and switch (no-change, blink, and eye reversal). The data of experiment II were analyzed for effects of percept (face or house), orientation (upright or inverted) and switch. For analyzing the frequency data the percept factor included the epochs where observers experienced ambiguous percepts. For analyzing the dominance durations, epochs with mixed percepts were not included. Correspondingly, and as commonly defined (Yu and Blake, 1992), we calculated the predominance ratio (*PR*) as the ratio of the summed dominance duration for one single stimulus alternative (e.d., *A*) to the sum of the added dominance durations of both rivaling stimulus alternatives (*A* + *B*)

(1) $$PR(\text{A})=\frac{\Sigma_{D}(\text{A})}{\Sigma_{D}(\text{A})+\Sigma_{D}(\text{B})},$$

hence *PR*(B) = 1 − *PR*(A). *PR* measures were calculated on the level of individual subjects, and were analyzed statistically.

In order to normalize differences in the mean duration of dominance epochs for the opponent rival stimuli we calculated a relative change measure *C*_%_ as

(2) $$C_{\%}=\frac{D_{A}-D_{B}}{D_{A}}\times 100\%.$$

where *A* was defined as the condition for which longer dominance epoch durations were expected, i.e., the upright orientation for rivalry of upright against inverted objects and the face category for rivalry of faces against houses.

## 3. Results

### 3.1. Frequencies of dominance epochs

**Figure 2:**
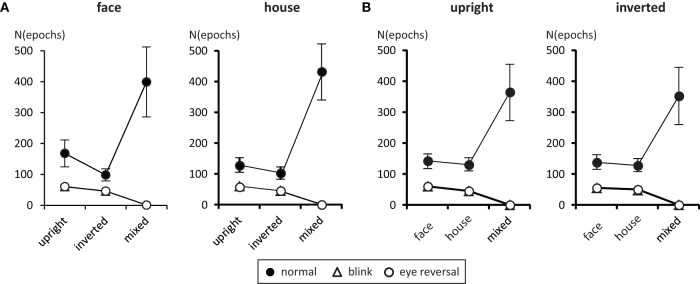
**Mean number of predominance epochs for upright faces and houses rivaling against their inverted counterparts (A), and faces rivaling against houses (B)**. Error bars indicate 95% confidence limits of the means.

Tables 1, 2 summarize the frequency statistics of the dominance epochs in the two experiments, and Figure 2 shows the mean number of epochs with their confidence intervals. In the no-change condition without eye reversal or blink the observers experienced about 665 dominance epochs for rivalry of faces and houses against their inverted counterparts, and for rivalry of faces against houses. In about half of all epochs (between 55% and 65%) the observers experienced “mixed” percepts, where they could not unambiguously decide between seeing alternative A or B. For the given stimulus size of about 3° visual angle, this result is in line with earlier findings (Yu and Blake, 1992). For the remaining epochs of unique percepts observers experienced a higher frequency of dominance epochs for upright than for inverted stimuli in experiment I (see Figure 2A). ANOVA revealed no overall effect of object type (face or house) [*F*_(1, 16)_ = 0.01, *p* = 0.941], an effect of percept [*F*_(2, 32)_ = 55.43, *p* < 0.001], and an interaction of percept with object type [*F*_(2, 32)_ = 13.46, *p* < 0.001], indicating a stronger effect of stimulus inversion for faces compared to houses. Pairwise comparisons within object category revealed inversion effects (calculated as the difference upright—inverted) for faces [*F*_(1, 16)_ = 26.13, *p* < 0.001] and for houses [*F*_(1, 16)_ = 18.85, *p* < 0.001].

**Table 1:** **Frequencies of dominance epochs for rivalry of upright versus inverted objects (*N* = 17)**.

	**No-change**				**Blink**				**Eye reversal**			
	**Face**		**House**		**Face**		**House**		**Face**		**House**	
Upright	168.1	25.3%	129.2	19.5%	60.5	56.5%	60.4	56.8%	60.4	56.2%	59.2	55.2%
Inverted	98.5	14.8%	102.7	15.5%	46.1	43.0%	45.1	42.4%	46.1	42.9%	46.4	43.3%
Mixed	398.9	59.9%	431.4	65.0%	0.5	0.4%	0.8	0.7%	0.9	0.9%	1.6	1.5%
Σ	665.5	100.0%	663.3	100.0%	107.1	100.0%	106.3	100.0%	107.4	100.0%	107.2	100.0%
*N* (faces)	879.9											
*N* (houses)	876.8											

**Table 2:** **Frequencies of dominance epochs for rivalry of faces versus houses (*N* = 17)**.

	**No-change**				**Blink**				**Eye reversal**			
	**Upright**		**Inverted**		**Upright**		**Inverted**		**Upright**		**Inverted**	
Face	142.1	22.2%	139.2	22.4%	59.5	56.0%	56.0	53.1%	62.8	58.7%	54.4	51.7%
House	131.5	20.6%	128.9	20.8%	46.1	43.3%	48.3	45.8%	43.2	40.4%	49.8	47.4%
Mixed	364.9	57.1%	352.5	56.8%	0.8	0.7%	1.2	1.1%	0.9	0.9%	0.9	0.8%
Σ	638.5	100.0%	620.6	100.0%	106.4	100.0%	105.5	100.0%	106.9	100.0%	105.1	100.0%
*N* (upright)	851.7											
*N* (inverted)	831.2											

For experiment II ANOVA indicated no overall effect of orientation [*F*_(1, 16)_ = 2.43, *p* = 0.138], an effect of percept [*F*_(2, 32)_ = 33.47, *p* < 0.001], and no interaction of percept with orientation [*F*_(2, 32)_ = 0.344, *p* = 0.711], substantiating the same pattern of effects in the two panels of Figure 2B. Pairwise comparisons within each orientation revealed no differences in the frequency of dominance epochs among the two rival objects in upright [*F*_(1, 16)_ = 0.77, *p* = 0.392] and inverted presentation [*F*_(1, 16)_ = 1.36, *p* = 0.261].

Tables 1, 2 validate that after data clearing blink and eye reversal epochs taken together still occurred with about the same frequency as the unique percepts in normal rivalry (i.e., the no-change condition). Eye reversal and blink trials did practically not occur during mixed percepts, since blink or eye reversal trials were initiated only when the subjects indicated prolonged unique dominance of one percept. Exceptions could occur only when the observer released a key precisely during the frame refresh before an eye reversal or switch. Such trials were excluded from the analyses.

### 3.2. Durations of dominance epochs

**Figure 3:**
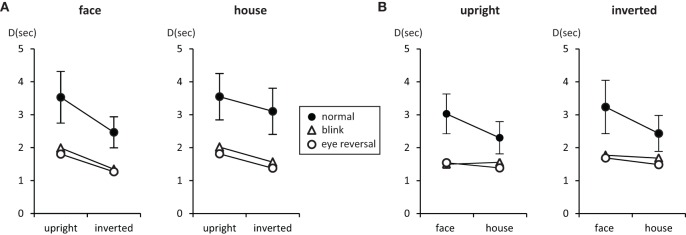
**Mean durations of dominance epochs (seconds) for upright faces and houses rivaling against their inverted counterparts (A), and faces rivaling against houses (B)**. Error bars indicate 95% confidence limits of the means.

Tables 3, 4 summarize the statistics for the average dominance durations of the two stimulus alternatives. The data are illustrated in Figure 3. For rivalry of upright against inverted objects (experiment I) ANOVA yielded main effects of percept [*F*_(1, 16)_ = 28.16, *p* < 0.001] and switch condition [*F*_(2, 32)_ = 43.32, *p* < 0.001], but no effect of object type [*F*_(1, 16)_ = 1.51, *p* = 0.236]. The object type × percept interaction failed significance [*F*_(1, 16)_ = 2.11, *p* = 0.165]. However, this result was due to the inclusion of the blink and eye reversal conditions. Analysis of just the data for normal, undisturbed rivalry epochs revealed a significant object type × percept interaction [*F*_(1, 16)_ = 6.51, *p* < 0.025], corresponding to the intersecting scheme of the means (see Figure 3A, solid symbols for faces and houses). The data in Table 3 show that the mean dominance times for upright faces were about 1000 ms longer than the mean dominance times for inverted faces, while the inversion effect for houses was less than 500 ms. The reduction of dominance time due to inversion (*C*_%_) was 30% for faces, compared to just 12.5% for houses in normal rivalry. Estimation of effect size for the inversion effects via the population variance estimates from the two paired samples (*d* = Δμ/$\hat{\sigma }$_*pop*_) revealed a large effect size (*d* > 0.8) for the inversion effect of faces, but a medium effect size for the inversion effect of houses (*d* ≈ 0.5), referring to Cohen's effect size classification (Cohen, 1988). Note that effects sizes for stimulus inversion in epochs with artificially induced termination (i.e., blink or eye reversal) yielded similar results (see Discussion).

**Table 3:** **Mean durations of dominance epochs (seconds) for rivalry of upright versus inverted objects (*N* = 17)**.

	**No-change**				**Blink**				**Eye reversal**			
	**Face**		**House**		**Face**		**House**		**Face**		**House**	
	**Upright**	**Inverted**	**Upright**	**Inverted**	**Upright**	**Inverted**	**Upright**	**Inverted**	**Upright**	**Inverted**	**Upright**	**Inverted**
Mean	3.529	2.464	3.549	3.104	1.989	1.344	2.018	1.561	1.804	1.267	1.813	1.378
*SE*	0.369	0.222	0.331	0.331	0.317	0.159	0.342	0.263	0.258	0.139	0.300	0.131
$\hat{\sigma }$_*pop*_	1.258		1.366		1.036		1.260		0.857		0.955	
Δ(*D*)	1.065		0.445		0.645		0.457		0.537		0.435	
Cohen's *d*	0.85		0.33		0.62		0.36		0.63		0.45	
*C*_%_	30.17		12.53		32.39		22.62		29.74		23.99	

**Table 4:** **Mean durations of dominance epochs (seconds) for rivalry of faces versus houses (*N* = 17)**.

	**No-change**				**Blink**				**Eye reversal**			
	**Upright**		**Inverted**		**Upright**		**Inverted**		**Upright**		**Inverted**	
	**Face**	**House**	**Face**	**House**	**Face**	**House**	**Face**	**House**	**Face**	**House**	**Face**	**House**
Mean	3.023	2.296	3.231	2.429	1.494	1.550	1.767	1.679	1.538	1.383	1.685	1.482
*SE*	0.285	0.230	0.382	0.257	0.207	0.199	0.325	0.264	0.229	0.153	0.332	0.219
$\hat{\sigma }$_*pop*_	1.069		1.343		0.840		1.222		0.805		1.161	
Δ(*D*)	0.727		0.802		−0.056		0.088		0.155		0.203	
Cohen's *d*	0.68		0.60		−0.07		0.07		0.19		0.17	
*C*_%_	24.04		24.84		−3.72		4.99		10.09		12.04	

For rivalry of faces against houses (experiment II) ANOVA indicated main effects of percept [*F*_(1, 16)_ = 10.37, *p* < 0.005] and switch condition [*F*_(2, 32)_ = 27.99, *p* < 0.001], but no effect of orientation [*F*_(1, 16)_ = 2.45, *p* = 0.136]. The orientation × percept interaction failed significance [*F*_(1, 16)_ = 1.01, *p* = 0.329]. This result persisted when analyzing the data for no-change conditions only [*F*_(1, 16)_ = 0.17, *p* = 0.689], corresponding to the parallel course of the means (see Figure 3B). Overall, the data demonstrate longer dominance durations for faces compared to houses independent of the orientation of the objects. The average difference in dominance duration was about 750 ms, which corresponds to 25% shorter dominance durations for houses compared to faces in the relative change measure, *C*_%_. Calculation of Cohen's *d* revealed a medium to large effect size of about *d* = 0.64 (see Table 4).

In both experiments the effects of blink and eye reversal practically coincided (see Figure 3). Both led to a strong shortening of the actual dominance epoch, having observers signal perceptual change approximately 750 ms after the manipulation occurred (see Tables 3, 4). Dominance of upright faces and houses apparently survived the manipulation for some extra time in experiment I (see Figure 3 and Discussion).

To check whether the results for the durations of the dominance epochs depend on the position on the duration scale we additionally analyzed the three quartiles of the dominance epoch duration distributions. Note that the dominance epoch durations usually follow a Gammy distribution (see Logothetis et al., 1996), which also holds for the data of this study for normal rivalry epochs which were not artificially terminated by blink or eye reversal (see distribution functions of dominance epoch durations in the electronic data supplement of this article). This means that, generally, Mod < Median < Mean holds for the duration data, so the distributions are positively skewed and the mean is the largest of all three distribution statistics, and is usually located between the median and the 3rd quartile. The results (see Supplementary Materials, Figure S1, and Tables S1, S2) show that the major findings obtained with the mean durations are maintained with all three quartiles: For normal rivalry there is an inversion effect of about 30% for faces, compared to just 12.5% for houses, and the duration advantage of faces over houses is about 25%. Also the effect sizes in the Cohen's *d* measure differ only marginally across the different duration statistics. This indicates that the effects of inversion and object category do not concern a particular band of epoch durations (e.g., only the longer ones), but all epoch durations to similar degrees. This is further indicated by the fact that the skewness of the distributions, measured via the third central moment, *m*_3_, is not modulated by inversion or object category in normal rivalry (see Table S3 in Supplementary Materials).

In order to get hints at possible response strategies in favor of upright objects (experiment I), or in favor of faces when rivaling against houses (experiment II), respectively, we analyzed the durations of the ambiguity epochs between the unique perceptual states (see Table S4 in Supplementary Materials). For experiment I the ambiguous epochs between the transition from upright to inverted objects and between the transition from inverted to upright objects had practically the same length [faces: Δ = 66 ms, *t*_16_ = −0.463, *p* = 0.649; houses: Δ = 63 ms, *t*_16_ = −0.554, *p* = 0.587]. However, for rivalry of faces against houses, the ambiguity epochs before the face percept were about 300–450 ms shorter than before the house percept [upright: Δ = 314 ms, *t*_(16)_ = −2.551, *p* < 0.05; inverted: Δ = 451 ms, *t*_(16)_ = −2.761, *p* < 0.05], indicating that subjects tended to resolve the ambiguity state earlier in favor of the face than the house percept. This may have perceptual or non-perceptual reasons (see Discussion).

### 3.3. Predominance ratios

The analyses in the foregoing sections has shown that upright objects gain an advantage in both the frequency of dominance epochs and their mean durations. Since the absolute dominance time of a perceptual alternative is given by the sum of durations of all its dominance epochs, the alternative which is more frequently dominant and has longer dominance periods will have larger absolute dominance time, and therefore show the larger predominance ratio (*PR*; see section 2). The Tables 5, 6 summarize the predominance ratios and their statistics for faces and houses rivaling against their inverted counterparts (Table 5) and faces rivaling against houses in upright and inverted orientation (Table 6). Using the *PR* the inversion effect is given by the deviation from the expected value *E*(*PR*) = 0.5 for equal absolute dominance durations (IE, last line of Tables 5, 6, listed in percent). A one sample test was calculated for the deviation of the *PR* from 0.5. The *PR* data from experiment I suggest significant inversion effects (*PR* > 0.5) for both faces and houses in all conditions. For normal rivalry (i.e., the no-change condition), the proportions of dominance times for upright and inverted objects were approximately 70:30 for faces, while, for houses, they were approximately 60:40. Calculation the odds ratio for the predominance ratios according to

(3) $$OR_{IE}(\text{face,house})=\frac{PR(\text{upright face})/(1-PR(\text{upright face}))}{PR(\text{upright house})/(1-PR(\text{upright house}))}$$

yielded a value of 1.59, indicating 1.6 times larger odds for upright faces compared to upright houses. For rivalry of faces against houses the *PR* values reveal dominance time proportions of approximately 60:40 in favor of faces (see Table 6) in normal rivalry, which is a significant deviation from an even distribution. This occurred for upright and inverted faces with approximately equal likelihood (*OR* = 1.04).

**Table 5:** **Predominance ratio statistics for rivalry of upright versus inverted objects (*N* = 17)**.

	**No-change**		**Blink**		**Eye reversal**	
	**Face**	**House**	**Face**	**House**	**Face**	**House**
Mean	0.696	0.590	0.648	0.631	0.644	0.595
*SE*	0.020	0.020	0.022	0.022	0.019	0.021
*t*	9.939	0.000	6.809	5.992	7.733	4.540
*p*	0.000	0.000	0.000	0.000	0.000	0.000
IE (%)	19.6	9.0	14.8	13.1	14.4	9.5

**Table 6:** **Predominance ratio statistics for rivalry of faces versus houses (*N* = 17)**.

	**No-change**		**Blink**		**Eye reversal**	
	**Upright**	**Inverted**	**Upright**	**Inverted**	**Upright**	**Inverted**
Mean	0.595	0.586	0.573	0.541	0.613	0.536
*SE*	0.024	0.030	0.031	0.030	0.020	0.020
*t*	3.906	2.896	2.396	1.340	5.588	1.819
*p*	0.001	0.011	0.029	0.199	0.000	0.088
IE (%)	9.5	8.6	7.3	4.1	11.3	3.6

## 4. Discussion

Measuring inversion effects for faces and houses in opponent-stimulus rivalry has revealed a strong advantage for upright objects. While inversion effects were found for both object categories, the effects for faces were significantly stronger, and involved both the frequency (see Table 1 and Figure 2A) and the mean duration of dominance epochs (see Table 3 and Figure 3A). Upright houses retained an advantage over inverted houses mostly with respect to mean epoch duration, and a smaller one in their frequency (ibid). The joint effect of frequency and duration of dominance epochs is impressive for faces, showing a distribution of 70:30 of total dominance time for upright faces rivaling against their inverted counterparts, compared to a 60:40 distribution for upright versus inverted houses. Moreover, the mean dominance duration advantage for upright faces of about 1 s, with an effect size of *d* = 0.85 is impressive, and contrasts strongly with the advantage of upright houses of scarcely half a second, amounting to an effect size of *d* = 0.33. The canonical result of experiment I is that both object categories show inversion effects in opponent-stimulus rivalry, but the effects for faces are disproportionately stronger. This means that both object classes are well separated with respect to their inversion effects in opponent-stimulus rivalry. The results of experiment II show that dominance epochs for faces and houses occur with equal frequency (see Figure 2B and Table 2), but the epochs of houses are about 25% shorter (see Table 4), leading to a 60:40 distribution of total dominance times for faces and houses independent of orientation. Overall, the results demonstrate that upright faces enjoy privileged presence in visual awareness.

We included blink and eye-reversal events in order to assemble evidence whether rivalry of common objects, which are known to be processed in specialized brain areas (Tong et al., 1998), rests more on eye- or pattern dominance (Blake et al., 1980; Logothetis et al., 1996). The most intriguing result found for these manipulations is that they yielded practically the same effect, namely terminating the current rivalry epoch. Dominance epochs in these conditions are about half as long as normal dominance epochs (see Figure 3 and Tables 3, 4), and their mean duration of about 1500 ms shows that these epochs terminate roughly 700–800 ms after the manipulation took effect. This is an expected delay caused by the evaluation of the changed percept and response preparation. If dominance rests on eye-specific mechanisms, immediate termination of the epoch is expected for the eye-reversal condition (Blake et al., 1980; Logothetis et al., 1996). However, since there is a local spatio-temporal luminance change caused by both eye reversal and blink, the termination of the current dominance epoch may be due to just this. A blink is merely a temporal disturbance of the same spatial image presentation while eye-reversal switches the eye-specific channels through which higher level object areas receive the stimulus input. Termination of their input should exert a greater effect than a brief interruption of the input flow in the same channels. In fact, it did not, regardless of the patterns which were rivaling. This points to pattern dominance (Logothetis et al., 1996) over eye dominance (Blake et al., 1980) for rivaling faces and houses. In further support of pattern dominance we observed inversion effects for faces and houses in these two conditions (see Figure 3A, and Tables 3, 5). Upright objects survived an eye reversal or blink for a longer time than their inverted counterparts, indicating that the termination of the dominance epoch is, at least partly, under higher level control, and not fully determined by the physical screen event.

The scheme of results for inversion effects reported here (experiment I) contrasts with effects found in continuous flash suppression (CFS), where a strong FIE was found, but no inversion effect for houses (Zhou et al., 2010). Stein et al. (2012) used CFS to study inversion effects for a large variety of objects. As in the present study a relative change measure was reported, which gauges the size of the effect independent of its absolute position on the time scale. For the *C*_%_ values, the authors obtained about 25% for faces, 20% for bodies, 6% for dogs and birds, and practically no effects for inanimate objects like lamps and chairs. Houses were not tested. In this study we obtained *C*_%_ values of about 30% for faces and 12.5% for houses. Although the data basis for the inversion effect in different binocular rivalry techniques is limited at the time, the superior inversion effects for faces and bodies in the study of Stein and colleagues indicate that CFS lets such objects reach visual awareness earlier which combine effects of familiarity and long-lasting learning (expertise) with the effects of domain-specific processing in specialized brain areas. Faces (FFA) and bodies (extrastriate body area (EBA) and fusiform body area (FBA; see Brandman and Yovel, 2010) were the only objects used in the study of Stein and colleagues that match both criteria. Houses only fit with the latter criterion (see Introduction), and fail to induce an inversion effect in CFS (Zhou et al., 2010). Findings of Jiang et al. (2007) point in the same direction. Using CFS they found strong inversion effects for faces and for Chinese and Hebrew words, but the latter only for readers of their own language.

In opponent-stimulus rivalry, where two unmasked and clearly visible stimulus alternatives compete for perceptual dominance, inversion effects are not limited to objects with domain specific processing and objects of expertise. Even for noisy dot figures that are more easily combined into meaningful objects under upright viewing conditions (Yu and Blake, 1992) the upright orientation is privileged. Moreover, the clear inversion effect obtained for houses in this study shows that in direct opponent-stimulus rivalry the upright view is preferred for those objects which are meaningful to us as common objects predominantly in upright orientation. We should therefore expect that plants, trees, chairs and lamps, which all failed to yield an inversion effect in CFS (Stein et al., 2012) yield inversion effects when paired in opponent-stimulus rivalry. The magnitudes of the inversion effects for faces and houses in opponent-stimulus rivalry resembles the magnitudes of inversion effects obtained for a variety of face and non-face objects in the seminal study on the effects of inversion by Yin (1969). The author compared recognition memory for photographs of faces with other objects which are mostly seen upright in everyday life (houses, airplanes, stickfigures). He obtained inversion effects for all objects, but recognition memory for faces was disproportionately impaired by inversion. This let him conclude that inversion effects reflect an experience dependent component that concerns all mono-oriented objects, as well as a component that is specific for faces. Apparently, both components shine through in direct opponent-stimulus rivalry, while in CFS only the latter component takes effect, comprising both generic category specific expertise (Carey and Diamond, 1977) and domain specificity (Kanwisher, 2000; Yovel and Kanwisher, 2004).

While studying inversion effects of the same stimuli in binocular rivalry is not confounded with low level image differences (experiment I), category specific effects (experiment II) are not easily evaluated. In this study we matched images for their 1st order luminance statistics, since images with larger contrasts are known to reduce the time of their suppression while their dominance times remain unchanged (Blake and Logothetis, 2002). We thus can assume that the 60:40 advantage for faces compared to houses is not due to different luminance histograms of both categories. Differences may, however, arise from category specific spatial frequency spectra. Control of amplitude spectra for face- and non-face objects is possible, but at the cost of a significant loss in face detail information (Willenbockel et al., 2010). Most current CFS studies on the inversion effect did not apply control of low level image properties, since they were not aiming at across category comparison of suppression times.

Results for opponent-stimulus rivalry show that particularly large inversion effects can be expected for faces, and minor but significant ones for other common mono-oriented objects. Hence, face speciality is well reflected by dominance in binocular rivalry. The large dominance advantage for upright faces makes the paradigm particularly suitable to study domains of face perception where the inversion effect is highly diagnostic, such as featural and relational image manipulations (Leder and Bruce, 2000; Leder et al., 2001), familiarity (Hancock et al., 2000; Veres-Injac and Persike, 2009), and own/other race effects (Young et al., 2012). Further, the smaller but present inversion effect for common mono-oriented objects renders them highly suitable as non-facial benchmarks. Inversion effects in CFS appear to be smaller and tightly focused on objects of expertise with domain specific processing. Hence, CFS exhibits higher categorial selectivity of the inversion effect.

A disadvantage of having observers track their perceptual states in opponent stimulus rivalry is that the tracking results may be confounded with possible response preferences, since subjects may tend to resolve ambiguous percepts earlier in favor of a preferred stimulus alternative. To account for possible response preferences, some authors use catch trials in which mixtures of both patterns overlayed in transparency, are presented to both eyes. A response bias in favor of one category is inferred from asymmetrical results in the dominance measure for the same mixture proportions, e.g., for 70:30 compared to 30:70 (Lee and Blake, 2004; Baker and Graf, 2009). Using this technique Baker and Graf (2009) found no evidence for a response tendency toward more familiar patterns when natural images rivaled against noise. We decided not to include such catch trials, since we already included the “blink” and “eye-reversal” trials, and interleaved binocular trials interfere with the dichoptic viewing cycle. However, analysis of the epochs with mixed percepts can give valuable hints whether possible response preferences might bias the subjects' perceptual reports. If such a bias exists, then the observers should signal the end of a mixed percept earlier when going from stimulus alternative A to B compared to moving from B to A. This means that, if there is a response bias toward one stimulus alternative, the mean durations of both kinds of mixed percepts should not be the same. The results (see Table S4 in Supplementary Materials) indicate same durations of the epochs with mixed percepts between upright and inverted objects and between inverted and upright objects, for both faces and houses. However, for rivalry of faces against houses, the mixed epochs that were resolved into faces were 300–450 ms shorter than the mixed epochs that ended up in houses, indicating a perceptual or a decisional asymmetry in the perceptual alternations among the object categories. On the basis of the present data it cannot be excluded that the observed face-to-house dominance ratio of 60:40 rests, at least partly, on response preferences for faces.

It is important to note that in opponent-stimulus rivalry observers just indicate what they actually see, and the stimulus alternatives are clearly visible and unmasked objects. In CFS, however, subjects perform a speeded detection task and the stimulus of interest is masked by a highly effective spatio-temporal noise masker. In view of the fact that there is external noise and decision noise in CFS it is not surprising that the influence of higher level stimulus properties, like structure and meaning, do not take effect so easily. However, CFS is much more apt for studying higher level stimulus influence on *unconscious* processing, including subcortical processing that may reach object-selective areas via subcortical projections (Pasley et al., 2004; Williams et al., 2004). Investigators may decide which paradigm applies best for the hypotheses under scrutiny.

## Author contributions

All authors contributed equally to conception and design of the study. Malte Persike conducted the experiments and data preparation. Günter Meinhardt contributed data analysis and interpretation. All authors were involved in writing, preparation of the manuscript and its final approval. All authors agree to be accountable for all aspects of the work in ensuring that questions related to the accuracy or integrity of any part of the work are appropriately investigated and resolved.

### Conflict of interest statement

The authors declare that the research was conducted in the absence of any commercial or financial relationships that could be construed as a potential conflict of interest.
