# Supplementary material for: The face inversion effect in opponent-stimulus rivalry
Source: Front Hum Neurosci. 2014 May 15;8:295. doi: 10.3389/fnhum.2014.00295 (PMC4030207; doi:10.3389/fnhum.2014.00295)
Supplement: Supplementary file 1 [file Presentation1.PDF]

The face inversion effect in opponent-stimulus rivalry

Malte Persike [1,*], Bozana Meinhardt-Injac [1] and Günter Meinhardt [1]

[1] *Department of Psychology, Johannes Gutenberg University Mainz, Germany*

Correspondence*:
Malte Persike
Department of Psychology, Methods Section, Johannes Gutenberg University Mainz, Wallstr. 3, 55099 Mainz, Germany, persike@uni-mainz.de

Facing the other: Novel theories and methods in face perception research

SUPPLEMENTARY MATERIAL: DATA ANALYSIS FOR THE QUARTILES OF THE DOMINANCE EPOCH DURATIONS AND SKEWNESS ANALYSIS

To complement the analysis based on the average dominance durations means we analyzed the three quartiles (i.e. the quantiles Q_{25}, Q_{50} (median) and Q_{75}). The Tables S1 and S2 show the significance levels of paired t- tests for upright versus inverted presentation (Tab. S1), and for faces rivaling against houses (Tab. S2). Further, the tables include Cohen's d and the relative change measure $C_{\%}$ for the mean and the three quartiles. Figure S1 shows the quartile data with their confidence intervals. Table S3 shows the results from the analysis of the 3rd central moment, m_3, for normal rivalry, and paired t- tests for this statistic for the four rivalry conditions.

Table S 1. Significance level of paired t-test, p, Cohen's d measure and relative change measure, $C_{\%}$, for inversion effects calculated from the mean, 3rd quartile (Q_{75}), median (Q_{50}), and the 1st quartile (Q_{25}) of the dominance epoch durations.

	normal		blink		eye reversal	
	face	house	face	house	face	house
$p(\overline{x})$	0.01	0.01	0.01	0.05	0.01	n.s.
$p(Q_{75})$	0.01	n.s.	0.05	0.05	0.05	n.s.
$p(Q_{50})$	0.01	0.01	0.05	0.01	0.05	n.s.
$p(Q_{25})$	0.01	0.05	n.s.	n.s.	n.s.	n.s.
$d(\overline{x})$	0.85	0.33	0.62	0.36	0.63	0.45
$d(Q_{75})$	0.90	0.22	0.52	0.44	0.54	0.52
$d(Q_{50})$	0.73	0.31	0.45	0.22	0.28	0.41
$d(Q_{25})$	0.69	0.36	0.29	0.11	0.24	0.18
$C_{\%}(\overline{x})$	30.17%	12.53%	32.39%	22.62%	29.74%	23.99%
$C_{\%}(Q_{75})$	30.50%	8.74%	31.48%	29.47%	30.05%	34.04%
$C_{\%}(Q_{50})$	27.25%	11.88%	24.66%	12.13%	15.53%	13.89%
$C_{\%}(Q_{25})$	29.12%	14.27%	14.40%	5.22%	11.97%	3.79%

Table S 2. Same statistics as shown in Tab. 7, but for rivalry of faces versus houses.

	normal		blink		eye reversal	
	upright	inverted	upright	inverted	upright	inverted
$p(\overline{x})$	0.01	0.01	n.s.	n.s.	n.s.	n.s.
$p(Q_{75})$	0.01	0.01	n.s.	n.s.	n.s.	n.s.
$p(Q_{50})$	0.01	0.01	n.s.	n.s.	n.s.	n.s.
$p(Q_{25})$	0.01	0.01	n.s.	n.s.	n.s.	n.s.
$d(\overline{x})$	0.68	0.60	-0.07	0.07	0.19	0.17
$d(Q_{75})$	0.70	0.65	-0.16	0.00	0.00	0.10
$d(Q_{50})$	0.77	0.53	-0.11	-0.01	0.00	0.09
$d(Q_{25})$	0.74	0.53	0.12	0.04	0.16	0.09
$C_{\%}(\overline{x})$	24.04%	24.84%	-3.72%	4.99%	10.09%	12.04%
$C_{\%}(Q_{75})$	24.81%	26.01%	-11.21%	-0.38%	-0.20%	8.45%
$C_{\%}(Q_{50})$	26.09%	22.16%	-6.34%	-1.18%	0.08%	6.88%
$C_{\%}(Q_{25})$	26.69%	24.99%	5.02%	3.10%	6.70%	5.58%

Table S 3. Third central moment, $m_3 = {}^1\!/\!_n \Sigma z^3$, for normal rivalry and paired t- test for this statistic for the four opponent-stimulus rivalry conditions ($N = 17$).

	face		house		upright		inverted	
	upright	inverted	upright	inverted	face	house	face	house
Mean	0.806	0.706	0.847	0.805	0.796	0.822	0.727	0.762
$\overline{\Delta}$	0.100		0.042		-0.026		-0.035	
$SE(\overline{\Delta})$	0.112		0.127		0.143		0.101	
t	0.888		0.328		-0.187		-0.348	
df	16		16		16		16	
p	0.388		0.747		0.854		0.732	

Table S4. Durations of the ambiguous rivalry epochs between the unique percepts of faces and houses for normal rivalry and paired t- test for the two orders of the perceptual transitions ($N = 17$).

	face		house		upright		inverted	
	$F \to iF$	$iF \to F$	$H \to iH$	$iH \to H$	$F \to H$	$H \to F$	$iF \to iH$	$iH \to iF$
Mean	1.278	1.344	1.407	1.470	1.255	0.941	1.532	1.081
$\overline{\Delta}$	-0.066		-0.063		0.314		0.451	
$SE(\overline{\Delta})$	0.142		0.114		0.123		0.163	
t	-0.463		-0.554		2.551		2.761	
df	16		16		16		16	
p	0.649		0.587		0.021		0.014	

FIGURES

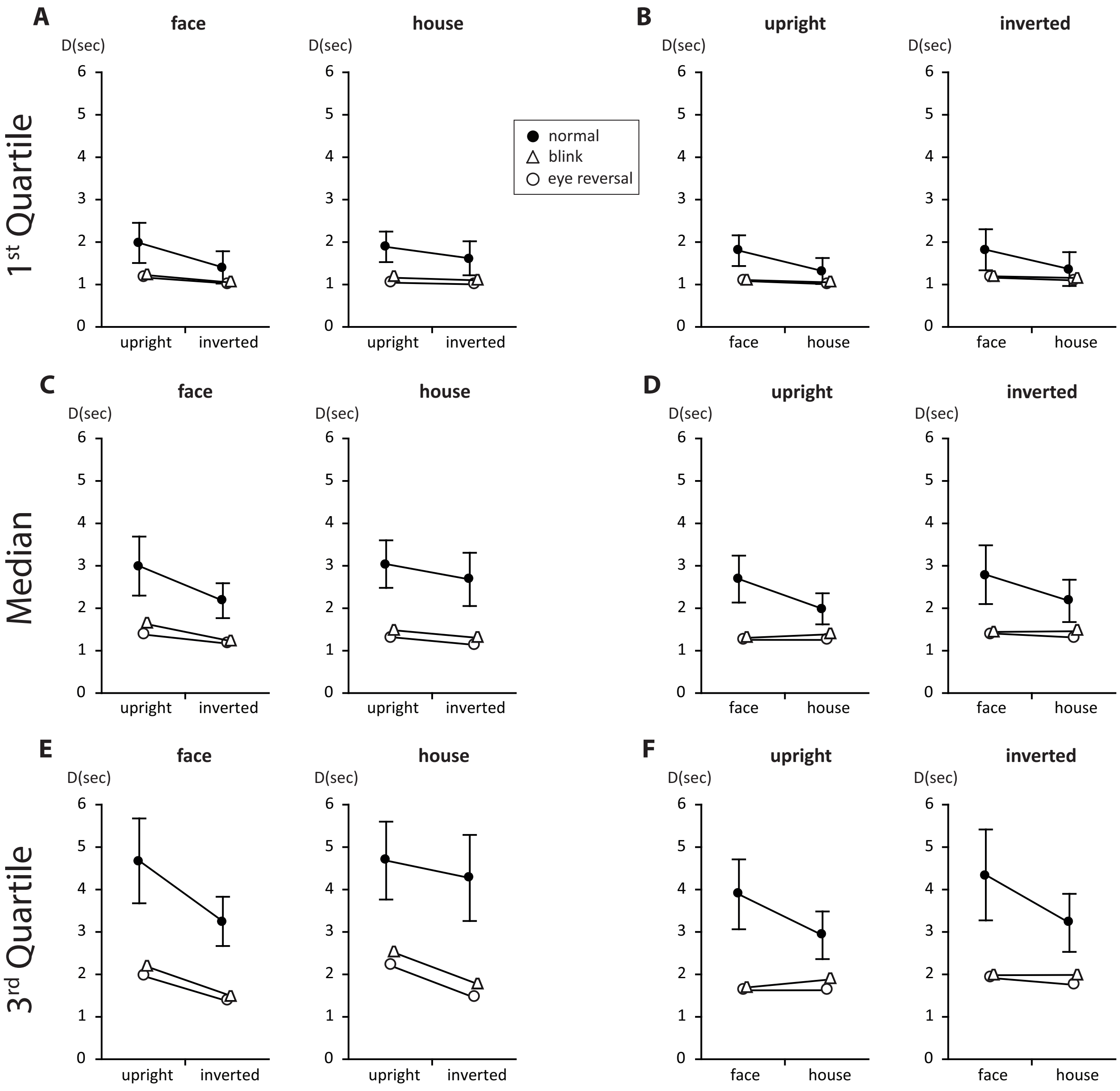

Figure S 1. Quartiles of the dominance epoch durations (sec.) for upright faces and houses rivaling against their inverted counterparts (left) and faces rivaling against houses (right). Error bars indicate 95% confidence limits of the measures.
